# Lack of resistance to macrolides in *Mycoplasma genitalium* detected in South African pregnant women

**DOI:** 10.4102/sajid.v36i1.209

**Published:** 2021-01-15

**Authors:** Meleshni Naicker, Ravesh Singh, Donald van der Westhuizen, Partson Tinarwo, Nathlee S. Abbai

**Affiliations:** 1 School of Clinical Medicine Research Laboratory, College of Health Sciences, Nelson R Mandela School of Medicine, University of KwaZulu-Natal, Durban, South Africa; 2 Department of Medical Microbiology, College of Health Sciences, Nelson R Mandela School of Medicine, University of KwaZulu-Natal, Durban, South Africa; 3 Department of Microbiology, National Health Laboratory Services, KwaZulu-Natal Academic Complex, Inkosi Albert Luthuli Central Hospital, Durban, South Africa; 4 Inqaba Biotechnical Industries (Pty) Ltd, Pretoria, South Africa; 5 Department of Biostatistics, College of Health Sciences, University of KwaZulu-Natal, Nelson R Mandela School of Medicine, Durban, South Africa; 6 School of Clinical Medicine Research Laboratory, Nelson R Mandela School of Medicine, University of KwaZulu-Natal, Durban, South Africa

**Keywords:** *Mycoplasma genitalium*, pregnant women, azithromycin, macrolide resistance, *23S rRNA* gene mutations, KwaZulu-Natal

## Abstract

**Background:**

Azithromycin regimens have been considered first-line treatment for *Mycoplasma genitalium* (*M. genitalium*), a sexually transmitted infection (STI) associated with adverse pregnancy outcomes. However, recent years have seen rapid emergence of macrolide resistance in *M. genitalium* as a result of widespread administration of azithromycin. Currently, there are limited data on macrolide resistance in pregnant women from KwaZulu-Natal (KZN), South Africa. This study investigated the prevalence of *M. genitalium* and emerging patterns of macrolide resistance in pregnant women from KZN.

**Methods:**

This was a sub-study of a larger study which involved laboratory-based detection of STIs in pregnant women. In the main study, pregnant women provided urine samples for detection of STIs. For this study, deoxyribose nucleic acid (DNA) extracted from stored urine was used to determine emerging macrolide resistance by amplification of the *23S ribosomal ribonucleic acid (rRNA)* gene of *M. genitalium* by polymerase chain reaction (PCR) and sequencing of amplicons to identify mutations associated with resistance. The Allplex™ MG & AziR assay was used as a confirmatory assay.

**Results:**

The prevalence of *M. genitalium* in pregnant women was 5.9% (13 out of 221). Sequencing of PCR amplicons did not reveal the presence of the A2059G and A2058G mutations associated with macrolide resistance. These findings were confirmed by the Allplex™ MG & AziR assay.

**Conclusion:**

Despite the lack of resistance to macrolides in this study population, continued antimicrobial resistance surveillance for *M. genitalium* in pregnant women is important because azithromycin is now part of the South African national STI syndromic management guidelines for vaginal discharge syndrome.

## Introduction

*Mycoplasma genitalium* (*M. genitalium*) is an emerging sexually transmitted infection (STI) that is associated with non-gonococcal urethritis (NGU) in men^1,2^ and cervicitis in women.^2,3^ In addition, genital mycoplasma infections in women have been implicated in various pathological conditions and upper genital tract complications during pregnancy, consequently giving rise to a host of adverse outcomes. These include pelvic inflammatory disease, endometritis, chorioamnionitis and postpartum fever, resulting in complications such as infertility, spontaneous abortion, stillbirth, preterm birth, low birth weight and perinatal mortality.^4,5,6,7,8,9^ However, there is still controversy regarding the role of *M. genitalium* in adverse pregnancy outcomes.^10,11,12^ Further, *M. genitalium* has been reported to be a possible contributing factor for the acquisition and transmission of human immunodeficiency virus (HIV).^13,14^ The prevalence of *M. genitalium* infection in women within the general population was reported between 1% and 4% but can reach 10% or higher in STI clinic attendees.^15,16,17^ In a study conducted in Pretoria, South Africa, in 2012 and 2016, the prevalence of *M. genitalium* in termination of pregnancy attendees was reported to be 6.0% in 2012 and 7.7% in 2016.^18^

Current South African Sexually Transmitted Infections Management Guidelines (2015) recommend the administration of a single oral dose (1 g) of azithromycin for the treatment of vaginal discharge syndrome and male urethritis syndrome.^19^ However, recent years have seen the rapid emergence of macrolide resistance in *M. genitalium* as a result of this widespread administration of azithromycin.^18,20,21,22,23,24,25^ The genetic basis for macrolide resistance was reported to be as a result of mutations occurring at positions 2058 and 2059 (*E. coli* numbering) within the V region of the *23S ribosomal ribonucleic acid (rRNA)* gene of *M. genitalium.*^22^

To date, there remains limited data available on the emergence of macrolide resistance in pregnant populations from KwaZulu-Natal (KZN). The present study provides data on the lack of macrolide resistance in pregnant women in our setting. In addition, this study provides data on the prevalence of *M. genitalium* in pregnant women, which is currently lacking in KZN.

## Materials and methods

### Study population and samples

The study was a sub-study of the larger study which involved laboratory-based detection of STIs in pregnant women (BE214/17). In the main study, pregnant women provided urine samples for detection of STIs. The women were recruited from the King Edward VIII Hospital in Durban, KZN, during the period November 2017 to April 2018. All enrolled women were 18 years and older, willing to provide written informed consent, information on their demographics, sexual behaviour, clinical information and a urine sample to be tested for vaginal infections.

Deoxyribose nucleic acid (DNA) was extracted from the urine using the PureLink^TM^ Microbiome DNA Purification Kit (ThermoFisher Scientific, Massachusetts, USA) in accordance with the manufacturer’s instructions. Unused DNA was stored at –20 °C. For this sub-study, *n* = 221 stored DNA samples were used to determine the presence of emerging resistance to macrolides amongst the participants.

### Detection of *Mycoplasma genitalium* from urine

Commercially available primers and probes specific for *M. genitalium* (Ba04646249_sl) were used with the TaqMan quantitative polymerase chain reaction (qPCR) assay (ThermoFisher Scientific, USA). The assay was run on the Quant Studio 5 Real-time PCR detection system (ThermoFisher Scientific, USA). The PCR was performed in a final reaction volume of 5 µL comprising: 0.25 µL Fluorescein amidite (FAM)-labelled probe and/or primer mix, 1.25 µL Fast Start 4x probe master mix (ThermoFisher, Part No. 4444434), 1.5 µL template DNA and nuclease-free water. Non-template control reactions and positive controls (TaqMan™ Vaginal Microbiota Extraction Control; cat no. A32039, ThermoFisher Scientific, USA) were also included. Amplification was performed at 95 °C for 30 s followed by 45 cycles comprising of denaturation at 95 °C for 3 s and annealing at 60 °C for 30 s. Detection of amplified fluorescent products was carried out at the end of the annealing phase. The raw fluorescent data included the C_T_ mean values, which were automatically generated by the Quant Studio 5 Real-time PCR system software (ThermoFisher Scientific, USA).

### Detection of mutations in the *23S ribosomal ribonucleic acid* gene conferring macrolide resistance

#### Screening assay: Conventional polymerase chain reaction

Macrolide resistance-associated mutations in the *23S rRNA* gene were determined for all samples that tested positive for *M. genitalium* by the TaqMan assay. The unique 147 base pair (bp) region within the *23S rRNA* gene of *M. genitalium*, which flanks mutations found in the V region of the gene, was amplified and sequenced.^22^ The screening PCR was performed in a final volume of 25 µL and comprised of 12.5 µL Dream Taq (2x) master mix (ThermoFisher Scientific, USA), 1 µL of each (10 µM) primer, 5 µL template DNA and nuclease-free water. Amplification was performed at an initial denaturation of 95 °C for 2 min, followed by 40 cycles of 95 °C for 30 s (denaturation), 54 °C for 1 min (annealing) and 72 °C for 1 min (extension). A final extension step at 72 °C for 5 min was included. Polymerase chain reaction amplicons were separated on 1% gel electrophoresis and visualised using an ultraviolet transilluminator (Gene Genius, SYNGENE, Maryland, USA).

#### Deoxyribose nucleic acid sequencing of amplicons

The 147 bp amplicon was sequenced using the Sanger method at Inqaba Biotechnological Industries in Pretoria, South Africa. The amplicons were sequenced using an ABI3500XL genetic analyser and the raw sequence data were edited using Chromas software V2.6.5 (Technelysium, Queensland, Australia) and then subjected to the National Centre for Biotechnology Information’s (NCBI) Basic Local Alignment Search Tool (BLAST) function for identity confirmation of the amplicons. This was followed by a multiple sequence alignment of study samples – V172, V193 and V208 – with strains of known mutations, LA141 (HF572938.1), LA088 (HF572933.1) and LA202 (HF572946.1), and a strain that does not include the mutations, that is, *M. genitalium* G37 complete genome (L43967.2), using ClustalW (https://www.genome.jp/tools-bin/clustalw).

#### *Confirmatory assay – Allplex™ MG & AziR assay* (Seegene)

The Allplex™ MG & AziR assay (Seegene, Seoul, South Korea) is a commercially available assay which allows for the simultaneous detection and identification of *M. genitalium* and six mutations *(A2058C, A2058G, A2058T, A2059C, A2059G* and *A2059T)* responsible for azithromycin resistance on validated sample types, which include genital swabs, urine and liquid-based cytology samples. The testing was performed as per the kit instructions and the samples were run on the Bio-Rad CFX96 equipped with Seegene interpretative software.

### Statistical analyses

The statistical analysis was conducted using R software (version 3.6.1), a freely available software environment for statistical computing. Initially, the population characteristics were described using frequencies stratified by infection status of the STI investigated. All the tests were conducted at 5% level of significance.

### Ethical consideration

Ethical approval to conduct the study was obtained from the Biomedical Research Ethics Committee (BREC), University of KwaZulu-Natal (UKZN), (reference no. BE214/17).

## Results

### Characteristics of the population according to *Mycoplasma genitalium* status

Out of 221 samples, 13 tested positive for *M. genitalium* using the TaqMan assay. The prevalence of *M. genitalium* in the study population was 5.9%. The median age of all 13 of the women who tested positive for *M. genitalium* was 26 years (interquartile range [IQR] 21–35). The analysis showed no significant associations (*p* > 0.05) between socio-demographic, behavioural and clinical data with reference to *M. genitalium* status (Table 1). Despite the lack of statistical significance, more than 60% of the women who tested *M. genitalium* positive did not present with symptoms of abnormal vaginal discharge on the day of enrolment. Similarly, more than 75% of the women did not experience previous symptoms of STIs 3 months prior to the enrolment. All women who tested positive for *M. genitalium* (*n* = 13) were unmarried. More than half of these women (53.8%) had reported having between two and four life-time sexual partners. A larger proportion of the positive women (53.8%) had indicated that either their current sexual partners had no other partners, or they were not aware of their partners having other partners. With regard to behavioural practices, 84.6% of the *M. genitalium* positive women had not used a condom during their last sexual act. Clinically, the majority of women who tested positive were in the third trimester of pregnancy (53.8%).

**TABLE 1:** Characteristics of the study population stratified by *Mycoplasma genitalium* status.

Characteristics	*Mycoplasma genitalium infection*												*p*
	Negative (*n* = 208)				Positive (*n* = 13)				Overall (*n* = 221)				
	*n*	%	Mean ± SD	Median	*n*	%	Mean ± SD	Median	*n*	%	Mean ± SD	Median	
**Age**	-	-		-	-	-	-	-	-	-	-	-	0.622
CV	-	21.1	28.3 ± 5.97	-	-	26.7	28.0 ± 7.47	-	-	21.3	28.3 ± 6.04	-	-
Q1	28	-	-	24.0	26	-	-	21.0	27	-	-	24.0	-
Q3	-	-	-	33.0	-	-	-	35.0	-	-	-	33.0	-
Minimum–maximum	18.0–43.0	-	-	-	20.0–42.0	-	-	-	18.0–43.0	-	-	-	-
**Current abnormal vaginal discharge**	-	-	-	-	-	-	-	-	-	-	-	-	0.771
No	136	65.4	-	-	8	61.5	-	-	144	65.2	-	-	-
Yes	72	34.6	-	-	5	38.5	-	-	77	34.8	-	-	-
**Symptoms of STIs in the past 3 months**	-	-	-	-	-	-	-	-	-	-	-	-	0.428
No	177	85.1	-	-	10	76.9	-	-	187	84.6	-	-	
Yes	31	14.9	-	-	3	23.1	-	-	34	15.4	-	-	
**Level of education**	-	-	-	-	-	-	-	-	-	-	-	-	0.139
Primary and below	13	06.2	-	-	2	15.4	-	-	15	06.8	-	-	-
High school	140	67.3	-	-	6	46.2	-	-	146	66.1	-	-	-
College/University	55	26.4	-	-	5	38.5	-	-	60	27.1	-	-	-
**Marital status**	-	-	-	-	-	-	-	-	-	-	-	-	0.223
No	175	84.5	-	-	13	100	-	-	188	85.5	-	-	-
Yes	32	15.5	-	-	0	0.0	-	-	32	14.5	-	-	-
**Has a regular sexual partner**	-	-	-	-	-	-	-	-	-	-	-	-	0.703
No	37	17.8	-	-	1	07.7	-	-	38	17.2	-	-	-
Yes	171	82.2	-	-	12	92.3	-	-	183	82.8	-	-	-
**Living with sexual partner**	-	-	-	-	-	-	-	-	-	-	-	-	0.24
No	126	60.6	-	-	10	76.9	-	-	136	61.5	-	-	-
Yes	82	39.4	-	-	3	23.1	-	-	85	38.5	-	-	-
**Age at first sex**	-	-	-	-	-	-	-	-	-	-	-	-	0.158
< 15	9	04.3	-	-	2	15.4	-	-	11	05.0	-	-	-
15–20	156	75.0	-	-	10	76.9	-	-	166	75.1	-	-	-
> 20	43	20.7	-	-	1	07.7	-	-	44	19.9	-	-	-
**Life-time number of sexual partners**	-	-	-	-	-	-	-	-	-	-	-	-	0.933
1	57	27.4	-	-	4	30.8	-	-	61	27.6	-	-	-
2–4	106	51.0	-	-	7	53.8	-	-	113	51.1	-	-	-
> 4	45	21.6	-	-	2	15.4	-	-	47	21.3	-	-	-
**Partner has other partners**	-	-	-	-	-	-	-	-	-	-	-	-	0.215
No/don’t know	148	71.2	-	-	7	53.8	-	-	155	70.1	-	-	-
Yes	60	28.8	-	-	6	46.2	-	-	66	29.9	-	-	-
**Condom use**	-	-	-	-	-	-	-	-	-	-	-	-	1
Never/rarely	72	34.6	-	-	4	30.8	-	-	76	34.4	-	-	-
Sometimes/always	136	65.4	-	-	9	69.2	-	-	145	65.6	-	-	-
**Condom use at last sexual act**	-	-	-	-	-	-	-	-	-	-	-	-	0.23
No	138	66.3	-	-	11	84.6	-	-	149	67.4	-	-	-
Yes	70	33.7	-	-	2	15.4	-	-	72	32.6	-	-	-
**Smokes**	-	-	-	-	-	-	-	-	-	-	-	-	0.091
No	201	96.6	-	-	11	84.6	-	-	212	95.9	-	-	-
Yes	7	03.4	-	-	2	15.4	-	-	9	04.1	-	-	-
**Consumes alcohol**	-	-	-	-	-	-	-	-	-	-	-	-	0.631
No	187	89.9	-	-	11	84.6	-	-	198	89.6	-	-	-
Yes	21	10.1	-	-	2	15.4	-	-	23	10.4	-	-	-
**Intravaginal practices**	-	-	-	-	-	-	-	-	-	-	-	-	1
No	188	90.4	-	-	12	92.3	-	-	200	90.5	-	-	-
Yes	20	09.6	-	-	1	07.7	-	-	21	09.5	-	-	-
**Trimester of pregnancy**	-	-	-	-	-	-	-	-	-	-	-	-	0.175
First	18	08.7	-	-	3	23.1	-	-	21	9.5	-	-	-
Second	72	34.6	-	-	3	23.1	-	-	75	33.9	-	-	-
Third	118	56.7	-	-	7	53.8	-	-	125	56.6	-	-	-
**Previous preterm delivery**	-	-	-	-	-	-	-	-	-	-	-	-	1
No	165	81.3	-	-	11	84.6	-	-	176	81.5	-	-	-
Yes	38	18.7	-	-	2	15.4	-	-	40	18.5	-	-	-
**Past miscarriage**	-	-	-	-	-	-	-	-	-	-	-	-	0.193
No	150	72.1	-	-	12	92.3	-	-	162	73.3	-	-	-
Yes	58	27.9	-	-	1	07.7	-	-	59	26.7	-	-	-
**Past spontaneous abortion**	-	-	-	-	-	-	-	-	-	-	-	-	1
No	189	90.9	-	-	12	92.3	-	-	201	91.0	-	-	-
Yes	19	9.1	-	-	1	07.7	-	-	20	09.0	-	-	-
**Previous abnormal vaginal discharge**	-	-	-	-	-	-	-	-	-	-	-	-	0.774
No	119	57.2	-	-	8	61.5	-	-	127	57.7	-	-	-
Yes	88	42.3	-	-	5	38.5	-	-	93	42.3	-	-	-
**Previously treated for STIs**	-	-	-	-	-	-	-	-	-	-	-	-	0.839
No	118	56.7	-	-	7	53.8	-	-	125	56.6	-	-	-
Yes	90	43.3	-	-	6	46.2	-	-	96	43.4	-	-	-

STI, sexually transmitted infection; CV, coefficient of variation.

### Detection of macrolide resistance-associated mutations in the *23S* ribosomal ribonucleic acid gene

#### Screening assay: Conventional polymerase chain reaction

**FIGURE 1:**
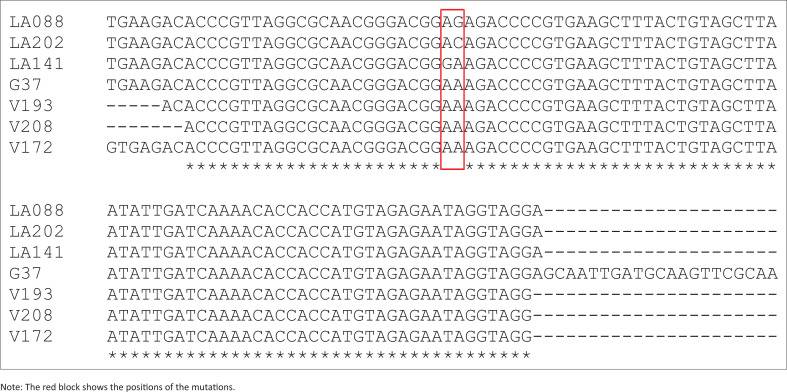
Multiple sequence alignment of study samples (V172, V193 and V208) with strains of known mutations, LA141 (HF 572938.1), LA088 (HF 572933.1) and LA202 (HF 572946.1), and strain G37, which lacks the mutations.

The 147 bp fragment associated with the *23S rRNA* gene was detected in 8 out of 13 (61.5%) of the *M. genitalium* positive isolates analysed. Sanger sequencing confirmed that the PCR amplicons obtained were the *23S rRNA* gene from *M. genitalium* (Accession number: Mk411362.1). Samples which produced good sequencing reads were aligned with strains with known mutations as well as a strain which lacked the mutations. According to the multiple sequence alignment, positions of known mutations associated with macrolide resistance, that is, A2058G, A2059C and A2059G, were not present in the study samples analysed. Similarly, these mutations were not present in the G37 strain (strain lacking the mutations) (Figure 1). However, as expected, the A2058G mutation was present in strain LA141, the A2059G mutation was present in strain LA088 and the A2059C mutation was present in strain LA202 (Figure 1). The prevalence of macrolide resistant *M. genitalium* in our study population was 0%.

#### Confirmatory assay – Allplex™ MG & AziR assay (Seegene)

The Allplex™ MG & AziR assay produced results for 11 out of 13 samples tested (84.61%) showing that it is much more sensitive than the screening assay (conventional PCR). The conventional PCR detected the *23S rRNA* in 61.5% of the samples tested (Table 1-A1). Two of the study samples V014 and V069 did not produce a positive result for the presence of *M. genitalium* on the Allplex™ MG & AziR assay despite testing positive on the TaqMan assay and yielding the 147 bp amplicon. The discrepancy between the assays may have been the result of loss of sample integrity (as a result of the freeze-thawing process) or loss of sample volume during shipment.

However, the Allplex™ MG & AziR assay confirmed that the prevalence of macrolide resistant *M. genitalium* in our study population was 0%. None of the study samples tested positive for any of the six mutations *(A2058C, A2058G, A2058T, A2059C, A2059G* and *A2059T)* responsible for azithromycin resistance (Table 2-A1). However, all assay controls yielded the expected results indicating that the results were valid.

## Discussion

To the best of our knowledge, this is the first study to provide an estimate on the prevalence of *M. genitalium* in pregnant women from the Durban area in KZN, South Africa. We report a prevalence estimate of 5.9% for *M. genitalium*. Our data are consistent with previous reports on *M. genitalium* infection in South African pregnant women.^18,26^ Redelinghuys et al. (2013) reported a prevalence of 14.5% for *M. genitalium* in pregnant women from Gauteng, South Africa.^26^ Similarly, another study conducted by Le Roux et al. (2018) reported a prevalence of 6.0% in 2012 and 7.7% in 2016 for *M. genitalium* in pregnant women from Pretoria, South Africa.^18^ In our study, the factors that could be attributed to this prevalence are as follows: 67.4% of the study women did not use condoms during their last sexual act; 61.5% were not living with their regular partner; 70.1% were unaware if their partner had other partners and 75.1% had first experienced sex between 15 and 20 years of age. In addition, 51.1% had between two and four life-time sex partners and 43.4% were previously treated for STIs. Many of these factors have been shown to contribute to the prevalence of sexually acquired infections.^27,28,29,30^

The emergence of macrolide resistance in *M. genitalium* has drawn recent attention.^18,20,21,22,23^ The first study to report on macrolide resistance in *M. genitalium* in South Africa was conducted by Hay et al. in the Limpopo province of rural South Africa in 2015.^20^ In that study, the authors reported detecting macrolide resistance-associated mutations in 4 out of 41 (9.8%) of *M. genitalium* positive isolates obtained from women attending a primary health care clinic. In contrast, in the current study, no macrolide resistance-associated mutations for the *M. genitalium* positive samples were reported. Our results are similar to that obtained in a more recent study conducted in Johannesburg, South Africa by Muller et al. (2019). In that study, no macrolide resistance-associated mutations were observed in their total study population of 266 stored *M. genitalium* positive isolates collected through the Gauteng STI National Microbiological Surveillance programme during the period 2007–2014.^21^ Similarly, Le Roux et al. (2018) reported no patterns of macrolide resistance amongst their *M. genitalium* positive isolates obtained in 2012 from pregnant women in Pretoria, South Africa.^18^ However, in that same study, the authors reported a macrolide resistance-associated mutation (A2059G) amongst their *M. genitalium* positive isolates obtained in 2016. The same mutation was present in 25% of their isolates tested. There is a possibility that the A2059G mutation is prevalent in South African populations; however, this needs further investigation to provide conclusive evidence.

Despite the lack of resistance to macrolides in the studied population, continued antimicrobial resistance surveillance for *M. genitalium* in pregnant women will be important since azithromycin is now part of the South African national STI syndromic management guidelines for vaginal discharge syndrome.

The present study was limited in that samples were collected from pregnant women attending a single antenatal facility. However, the hospital from which the women were sampled in this study serves as a central hospital for women from around the Durban area, thereby making the population more generalised. The strength of this study is that we now provide prevalence data as well as data on macrolide susceptibility of *M. genitalium* in a population of pregnant women from KZN, an area of research that has received limited attention.

## Appendix 1

**TABLE 1-A1:** Data from the sensitivity analysis of the 23S ribosomal ribonucleic acid conventional polymerase chain reaction when compared to the AziR assay (comparator assay) computed using R software (version 3.6.1).

AziR assay (comparator assay): Explanation	23S PCR			
	Element	Estimate	Lower	Upper
Apparent (test or predicted) prevalence (+)	APREV	0.615	0.316	0.861
True (outcome) prevalence (+)	TPREV	0.846	0.546	0.981
Sensitivity (correct [+] in diseased)	SE	0.545	0.234	0.833
Specificity (correct [−] in not diseased)	SP	0.000	0.000	0.842
Diagnostic accuracy (total correct [+] and [−])	DIAG.ACC	0.462	0.192	0.749
Diagnostic OR (likely correct [+] in diseased)	DIAG.OR	0.000	0.000	-
Number needed to diagnose (to give one [+])	NND	−2.200	−1.305	1.483
Youden’s index (max true [+] without false [+])	YOUDEN	−0.455	−0.766	0.674
PPV (genuinely diseased in [+])	PPV	0.750	0.349	0.968
NPV (genuinely not diseased in [−])	NPV	0.000	0.000	0.522
LR+ (true [+] to one false [+])	PLR	0.545	0.318	0.936
LR- (false [−] to 10 true [−])	NLR	-	-	-
Proportion of subjects with the outcome ruled out	PRO	0.385	0.139	0.684
Proportion of subjects with the outcome ruled in	PRI	0.615	0.316	0.861
Proportion of false (+)	PFP	1.000	0.158	1.000
Proportion of false (−)	PFN	0.455	0.167	0.766
Agreement beyond one expected by chance alone	KAPPA	−0.282	−0.736	0.173
Dichotomous correlation	PHI	−0.200	-	-
Equivalent continuous scale correlation	TETRACHORIC	−0.386	-	-
Non and diseased discrimination ability (z-score)	D’PRIME	−0.727	-	-
Human interpretation bias	CRITERION	−0.842	-	-
Average sensitivity for all specificity values	AUC	0.304	-	-
Test for presence of bias (systematic difference)	MCNEMAR	*p* = 0.257	-	-

PCR, polymerase chain reaction; PPV, positive predictive value; NPV, negative predictive value; APREV, apparent prevalence; TPREV, true prevalence; DIAG.ACC, diagnostic accuracy; DIAG.OR, diagnostic odds ratio; PLR, positive log ratio; NLR, negative log ratio; SE, sensitivity; SP, specificity; NND, number needed to diagnose; PRO, proportion ruled out; PRI, proportion ruled in; PFP, proportion of false positives; PFN, proportion of false negatives; AUC, Area under the curve.

**TABLE 2-A1:** Data obtained for the clinical samples using the Allplex™ MG & AziR assay (Seegene): Output obtained directly from the Seegene viewer linked to the Bio-Rad CFX96 instrument.

Well	Name	Type	FAM				HEX				Cal Red 610				Quasar 670		Quasar 670		Auto Interpretation
			A2059T	C(t)	A2058T	C(t)	A2058C	C(t)	A2058G	C(t)	A2059C	C(t)	A2059G	C(t)	MG	C(t)	IC	C(t)	
A01	V014	SAMPLE	−	N/A	−	N/A	−	N/A	−	N/A	−	N/A	−	N/A	−	N/A	+	29.32	−
B01	V019	SAMPLE	−	N/A	−	N/A	−	N/A	−	N/A	−	N/A	−	NIA	+	37.12	+	26.19	MG
C01	V055	SAMPLE	−	N/A	−	N/A	−	N/A	−	N/A	−	N/A	−	NIA	+	34.90	+	26.72	MG
D01	V069	SAMPLE	−	N/A	−	N/A	−	N/A	−	N/A	−	N/A	−	NIA	−	N/A	+	28.60	−
E01	V083	SAMPLE	−	N/A	−	N/A	−	N/A	−	N/A	−	N/A	−	NIA	+	35.71	+	28.31	MG
F01	V103	SAMPLE	−	N/A	−	N/A	−	N/A	−	N/A	−	N/A	−	NIA	+	32.07	+	25.13	MG
G01	V104	SAMPLE	−	N/A	−	N/A	−	N/A	−	N/A	−	N/A	−	NIA	+	30.11	+	25.00	MG
H01	V109	SAMPLE	−	N/A	−	N/A	−	N/A	−	N/A	−	N/A	−	NIA	+	34.57	+	25.64	MG
A12	V193	SAMPLE	−	N/A	−	N/A	−	N/A	−	N/A	−	N/A	−	NIA	+	26.33	+	25.07	MG
B12	V172	SAMPLE	−	N/A	−	N/A	−	N/A	−	N/A	−	N/A	−	NIA	+	26.25	+	22.96	MG
C12	V197	SAMPLE	−	N/A	−	N/A	−	N/A	−	N/A	−	N/A	−	NIA	+	37.96	+	22.97	MG
D12	V208	SAMPLE	−	N/A	−	N/A	−	N/A	−	N/A	−	N/A	−	NIA	+	26.50	+	23.59	MG
E12	V238	SAMPLE	−	N/A	−	N/A	−	N/A	−	N/A	−	N/A	−	NIA	+	35.29	+	27.30	MG
F12	PC	PC	+	26.75	+	23.07	+	23.46	+	22.59	+	23.05	+	22.13	+	20.20	+	24.70	Positive Control(+)
G12	NC	NC	−	N/A	−	N/A	−	N/A	−	N/A	−	N/A	−	N/A	−	N/A	−	N/A	Negative Control(−)

N/A, not applicable; FAM, fluorescein amidite; HEX, hexachlorofluorescein; PS, positive control; NC, negative control.
